# Evaluation of Temporal Trends in Racial and Ethnic Disparities in Sleep Duration Among US Adults, 2004-2018

**DOI:** 10.1001/jamanetworkopen.2022.6385

**Published:** 2022-04-07

**Authors:** César Caraballo, Shiwani Mahajan, Javier Valero-Elizondo, Daisy Massey, Yuan Lu, Brita Roy, Carley Riley, Amarnath R. Annapureddy, Karthik Murugiah, Johanna Elumn, Khurram Nasir, Marcella Nunez-Smith, Howard P. Forman, Chandra L. Jackson, Jeph Herrin, Harlan M. Krumholz

**Affiliations:** 1 Center for Outcomes Research and Evaluation, Yale New Haven Hospital, New Haven, Connecticut; 2 Section of Cardiovascular Medicine, Department of Internal Medicine, Yale School of Medicine, New Haven, Connecticut; 3 Section of General Internal Medicine, Department of Internal Medicine, Yale School of Medicine, New Haven, Connecticut; 4 Division of Cardiovascular Prevention and Wellness, Houston Methodist DeBakey Heart and Vascular Center, Houston, Texas; 5 Center for Outcomes Research, Houston Methodist Research Institute, Houston, Texas; 6 Department of Chronic Disease Epidemiology, Yale School of Public Health, New Haven, Connecticut; 7 Department of Pediatrics, University of Cincinnati College of Medicine, Cincinnati, Ohio; 8 Division of Critical Care Medicine, Cincinnati Children’s Hospital Medical Center, Cincinnati, Ohio; 9 SEICHE Center for Health and Justice, Section of General Internal Medicine, Yale School of Medicine, New Haven, Connecticut; 10 Equity Research and Innovation Center, Section of General Internal Medicine, Yale School of Medicine, New Haven, Connecticut; 11 Department of Radiology and Biomedical Imaging, Yale School of Medicine, New Haven, Connecticut; 12 Epidemiology Branch, National Institute of Environmental Health Sciences, National Institutes of Health, Department of Health and Human Services, Research Triangle Park, North Carolina; 13 Intramural Program, National Institute on Minority Health and Health Disparities, National Institutes of Health, Department of Health and Human Services, Bethesda, Maryland; 14 Department of Health Policy and Management, Yale School of Public Health, New Haven, Connecticut

## Abstract

**Importance:**

Historically marginalized racial and ethnic groups are generally more likely to experience sleep deficiencies. It is unclear how these sleep duration disparities have changed during recent years.

**Objective:**

To evaluate 15-year trends in racial and ethnic differences in self-reported sleep duration among adults in the US.

**Design, Setting, and Participants:**

This serial cross-sectional study used US population-based National Health Interview Survey data collected from 2004 to 2018. A total of 429 195 noninstitutionalized adults were included in the analysis, which was performed from July 26, 2021, to February 10, 2022.

**Exposures:**

Self-reported race, ethnicity, household income, and sex.

**Main Outcomes and Measures:**

Temporal trends and racial and ethnic differences in short (<7 hours in 24 hours) and long (>9 hours in 24 hours) sleep duration and racial and ethnic differences in the association between sleep duration and age.

**Results:**

The study sample consisted of 429 195 individuals (median [IQR] age, 46 [31-60] years; 51.7% women), of whom 5.1% identified as Asian, 11.8% identified as Black, 14.7% identified as Hispanic or Latino, and 68.5% identified as White. In 2004, the adjusted estimated prevalence of short and long sleep duration were 31.4% and 2.5%, respectively, among Asian individuals; 35.3% and 6.4%, respectively, among Black individuals; 27.0% and 4.6%, respectively, among Hispanic or Latino individuals; and 27.8% and 3.5%, respectively, among White individuals. During the study period, there was a significant increase in short sleep prevalence among Black (6.39 [95% CI, 3.32-9.46] percentage points), Hispanic or Latino (6.61 [95% CI, 4.03-9.20] percentage points), and White (3.22 [95% CI, 2.06-4.38] percentage points) individuals (*P* < .001 for each), whereas prevalence of long sleep changed significantly only among Hispanic or Latino individuals (−1.42 [95% CI, −2.52 to −0.32] percentage points; *P* = .01). In 2018, compared with White individuals, short sleep prevalence among Black and Hispanic or Latino individuals was higher by 10.68 (95% CI, 8.12-13.24; *P* < .001) and 2.44 (95% CI, 0.23-4.65; *P* = .03) percentage points, respectively, and long sleep prevalence was higher only among Black individuals (1.44 [95% CI, 0.39-2.48] percentage points; *P* = .007). The short sleep disparities were greatest among women and among those with middle or high household income. In addition, across age groups, Black individuals had a higher short and long sleep duration prevalence compared with White individuals of the same age.

**Conclusions and Relevance:**

The findings of this cross-sectional study suggest that from 2004 to 2018, the prevalence of short and long sleep duration was persistently higher among Black individuals in the US. The disparities in short sleep duration appear to be highest among women, individuals who had middle or high income, and young or middle-aged adults, which may be associated with health disparities.

## Key Points

**Question:**

How have racial and ethnic differences in self-reported sleep duration among US adults changed from 2004 to 2018?

**Findings:**

In this cross-sectional study of 429 195 US adults, the prevalence of short and long sleep duration were persistently higher among Black individuals during the 15-year study period. The disparities in short sleep duration were highest for Black women, Black individuals with middle or high income, and young and middle-aged Black adults.

**Meaning:**

These findings suggest that marked racial and ethnic differences in sleep duration persisted from 2004 to 2018 and may contribute to health disparities among Black individuals.

## Teaser

This cross-sectional study describes the temporal trends in racial and ethnic disparities in sleep duration during a 15-year period among US adults with sleep data available from the National Health Interview Survey.

## Introduction

In the US, historically marginalized racial and ethnic groups are generally more likely to report and experience sleep deficiencies that may be drivers of racial and ethnic disparities in physical health, mental health, and quality of life.^1,2,3,4,5,6,7,8,9^ Both short and long sleep duration are more prevalent among Black and Hispanic or Latino individuals compared with White individuals.^10,11,12,13,14^ The proportion of people reporting short sleep duration has increased across different racial and ethnic groups, widening the gap between Black and White individuals in recent years.^12^ This increase occurred while a national health objective to increase the proportion of people with sufficient sleep was in place.^15,16^

In 2020, the National Institute on Minority Health and Health Disparities; the National Heart, Lung, and Blood Institute; and the Office of Behavioral and Social Sciences Research proposed a framework for sleep health disparities research that focused on the need for greater understanding of health consequences and interventions that may eliminate them.^8^ Their report underscores the need for a more detailed evaluation of the population-level trends in sleep health disparities.

Several key gaps in knowledge persist. First, although information on short sleep is available, our understanding of trends in disparities in long sleep, which is also a risk factor for adverse health outcomes, remains poor. Moreover, little information is available on trends in the racial and ethnic disparities in sleep health stratified by age, sex, or household income.^17,18,19^ For instance, an understanding of how racial and ethnic differences in sleep duration vary with age may illuminate the periods during a lifetime in which these disparities emerge and peak. Furthermore, there are known differences in sleep duration by age and sex,^20^ and people with low income are more likely to report poorer sleep health.^21^ How these differences vary by race and ethnicity remains unknown, and a deeper understanding of these variations is important to identify at-risk groups and institute effective interventions. Finally, many studies have not included Asian individuals as a distinct albeit heterogeneous racial group.

Accordingly, we evaluated the temporal trends in racial and ethnic disparities in sleep duration during a 15-year period using representative data from the National Health Interview Survey (NHIS). We estimated differences in the reported short or long sleep duration between racial and ethnic groups overall and stratified by sex, household income, and health status. In addition, we evaluated the racial and ethnic differences in the association between sleep duration and age. The purpose of this study is to illuminate trends in racial and ethnic disparities in sleep duration and thereby inform policies and practices designed to address these disparities.

## Methods

### Data Source

We used data from the annual NHIS from 2004 to 2018. The NHIS has a complex multistage area probability design that accounts for nonresponse and oversampling of underrepresented groups, which allows for nationally representative estimates (details in eMethods in the Supplement).^22^ We used data from the Sample Adult Core file, which includes responses from 1 randomly selected adult from each family for a more in-depth questionnaire (mean conditional response rate from 2004 to 2018, 80.3%; mean final response rate from 2004 to 2018, 62.1%) (eMethods in the Supplement). We obtained the harmonized data from the Integrated Public Use Microdata Series Health Surveys,^23^ including the NHIS strata, primary sampling unit, and person weights. All NHIS respondents provided oral consent before participation. The institutional review board at Yale University exempted the study from review because NHIS data are publicly available. The study adhered to the Strengthening the Reporting of Observational Studies in Epidemiology (STROBE) reporting guideline.

### Study Population

We included individuals 18 years or older from 2004 to 2018 of the NHIS. We excluded respondents with missing sleep data. Owing to small numbers, we also excluded those who identified as non-Hispanic Alaskan Native or American Indian and those who identified as non-Hispanic and did not select a primary race (details are given in the Results section).

### Demographic Variables

Participants were classified into 4 mutually exclusive racial and ethnic subgroups—non-Hispanic Asian (hereinafter, Asian), non-Hispanic Black or African American (hereinafter, Black), Hispanic or Latino, and non-Hispanic White (hereinafter, White)—based on their responses to the following questions: “What race do you consider yourself to be?” and “Do you consider yourself Latino/Hispanic?” Other characteristics included were self-reported age, sex, household income level, health status, and geographic region. Based on the family income level relative to the respective year’s federal poverty level from the US Census Bureau, income level was categorized as low (<200% of the federal poverty level) or middle to high (≥200% of the federal poverty level).^24,25,26^ Other clinical and sociodemographic characteristics were used only to describe the study population (eMethods in the Supplement).

### Sleep Duration

In the NHIS, participants were asked, “On average, how many hours of sleep do you get in a 24-hour period?” The responses were coded as integers, rounded to the nearest hour (eMethods in the Supplement). We defined recommended sleep duration as 7 to 9 hours of sleep in a 24-hour period, short sleep duration as fewer than 7 hours, and long sleep duration as more than 9 hours, consistent with expert consensus recommendations.^27^

### Statistical Analysis

We first described the general characteristics of the study population. For each year, we estimated the short and long sleep duration prevalence for each racial and ethnic group using multivariable multinomial logistic regression models, adjusting for age and region (details are provided in the eMethods in the Supplement). To measure the racial and ethnic differences in short and long sleep duration, we subtracted the annual prevalence among White individuals from the annual prevalence among Asian, Black, and Hispanic or Latino individuals for that year. Using these annual estimates and differences between estimates, we determined trends during the study period by fitting weighted linear regression models. In a separate analysis, we tested for an absolute difference in each sleep duration prevalence within each racial and ethnic group and the differences between groups from 2004 to 2018 using a *z* test.

To evaluate the association between race and ethnicity and each of these sleep duration outcomes by age, we used multinomial logistic regression models with categorical sleep duration as the dependent variable and age group as the independent variable (eMethods in the Supplement). We then stratified the main analysis described above by sex and household income separately. Owing to the high amount of missing income information from nonresponse, the NHIS data include multiply imputed income variables for respondents who do not report income. Thus, our income-stratified analysis was performed based on the National Center for Health Statistics recommendations for multiply imputed data analysis (eMethods in the Supplement).^28^ For a supplementary analysis, we also stratified the main temporal trends analysis by health status to explore the extent to which the sleep disparities were explained by racial and ethnic differences in self-perceived health. Finally, we performed a sensitivity analysis to assess whether the observed disparities in short sleep duration between Black and White individuals may be explained solely by differences in self-report bias of sleep duration (eMethods in the Supplement).^29^

For all analyses, a 2-sided *P* < .05 was used to determine statistical significance. All analyses were performed between July 26, 2021, and February 10, 2022, using Stata SE, version 17.0 (StataCorp LLC), and incorporated the NHIS strata, primary sampling unit, and sample adult weights to produce nationally representative estimates using the svy family of commands for structured survey data. All results are reported with 95% CIs. The NHIS strata, primary sampling unit, and person weights were obtained from the Integrated Public Use Microdata Series. All person weights were pooled and divided by the number of years studied according to guidance from the NHIS.^30^

## Results

### Population Characteristics

Among the 444 743 adults interviewed from 2004 to 2018, we excluded 10 203 (2.3%) who had missing information on sleep duration. Because of small numbers, we also excluded 3440 individuals who identified as non-Hispanic Alaskan Native or American Indian and 1905 individuals who identified as non-Hispanic and did not select a primary race (eFigure 1 in the Supplement). Thus, the study sample consisted of 429 195 individuals (median [IQR] age, 46 [31-60] years; 51.7% [95% CI, 51.5%-51.9%] women and 48.3% [95% CI, 48.1%-48.5%] men), of whom 5.1% (95% CI, 4.9%-5.2%) identified as Asian, 11.8% (95% CI, 11.5%-12.2%) identified as Black, 14.7% (95% CI, 14.2%-15.1%) identified as Hispanic or Latino, and 68.5% (95% CI, 67.9%-69.0%) identified as White. Study population characteristics are shown in Table 1, and the unadjusted sleep duration distribution by race and ethnicity is shown in eFigure 2 in the Supplement.

**Table 1.** Study Population Characteristics

Characteristic	Participant race and ethnicity^a^				
	Asian (n = 22 924)	Black (n = 61 226)	Hispanic or Latino (n = 71 567)	White (n = 273 478)	All (N = 429 195)
Age, median (IQR), y	42 (31-55)	42 (29-56)	38 (28-51)	48 (33-62)	46 (31-60)
Age group, y					
18-39	44.6 (43.5-45.6)	44.8 (44.1-45.5)	53.7 (53.0-54.3)	34.5 (34.0-34.9)	39.0 (38.6-39.4)
40-64	42.1 (41.2-43.0)	42.2 (41.7-42.8)	37.2 (36.7-37.7)	44.7 (44.4-45.1)	43.2 (42.9-43.5)
≥65	13.4 (12.7-14.0)	12.9 (12.5-13.3)	9.1 (8.8-9.5)	20.8 (20.5-21.1)	17.8 (17.5-18.0)
Sex					
Women	52.8 (52.0-53.6)	55.0 (54.5-55.6)	49.3 (48.8-49.8)	51.6 (51.4-51.8)	51.7 (51.5-51.9)
Men	47.2 (46.4-48.0)	45.0 (44.4-45.5)	50.7 (50.2-51.2)	48.4 (48.2-48.7)	48.3 (48.1-48.5)
US citizenship (n = 428 343)	69.7 (68.5-70.8)	95.3 (94.9-95.6)	65.2 (64.3-66.1)	98.5 (98.4-98.5)	91.8 (91.5-92.0)
Educational level attained (n = 426 934)					
Less than high school	9.4 (8.8-10.1)	16.8 (16.3-17.3)	35.3 (34.5-36.0)	9.5 (9.3-9.7)	14.1 (13.8-14.4)
High school diploma/GED	26.8 (26.5-27.1)	30.3 (29.7-30.9)	26.5 (26.0-27.0)	26.8 (26.5-27.1)	26.6 (26.4-26.9)
Some college	22.3 (21.4-23.1)	33.3 (32.7-34.0)	25.0 (24.4-25.5)	31.4 (31.1-31.7)	30.2 (30.0-60.5)
Bachelor’s degree or higher	32.3 (31.8-32.8)	19.6 (19.0-20.2)	13.3 (12.8-13.7)	32.3 (31.8-32.8)	29.1 (28.6-29.5)
Annual income <200% federal poverty level^b^	28.0 (24.9-31.3)	46.4 (44.1-48.7)	51.4 (49.4-48.7)	24.0 (23.0-25.0)	30.8 (29.9-31.7)
Uninsured at the time of interview (n = 427 762)	12.0 (11.4-12.6)	18.1 (17.6-18.6)	33.4 (32.6-34.2)	10.3 (10.1-10.6)	14.7 (14.5-15.0)
US region^c^					
Northeast	19.7 (18.4-21.2)	15.9 (15.1-16.8)	13.7 (12.8-14.6)	18.9 (18.4-19.5)	17.8 (17.4-18.3)
Midwest	13.1 (12.0-14.2)	17.5 (16.6-18.5)	9.2 (8.3-10.0)	28.0 (27.3-28.7)	23.2 (22.7-23.8)
South	22.0 (20.6-23.5)	58.2 (56.8-59.6)	36.6 (35.1-38.1)	33.8 (33.0-34.5)	36.5 (35.8-37.1)
West	45.2 (43.3-47.1)	8.4 (7.9-8.9)	40.6 (39.0-42.2)	19.4 (18.8-20.0)	22.5 (22.0-23.1)
Married or living with partner (n = 427 923)	64.5 (63.5-65.5)	34.5 (33.9-35.1)	53.0 (52.4-53.6)	57.8 (57.3-58.2)	54.7 (54.3-55.0)
Employment status (n = 428 865)					
With a job/working	65.1 (64.2-66.0)	59.9 (59.2-60.5)	65.3 (64.7-65.9)	62.3 (61.9-62.7)	62.6 (62.3-62.9)
Not in labor force	30.8 (29.9-31.7)	31.9 (31.2-32.5)	29.1 (28.5-29.7)	34.3 (33.9-34.6)	33.0 (32.7-33.4)
Unemployed	4.1 (3.8-4.4)	8.3 (8.0-8.6)	5.7 (5.4-5.9)	3.5 (3.4-3.6)	4.4 (4.3-4.5)
Poor or fair health	8.9 (8.4-9.4)	17.7 (17.3-18.2)	13.8 (13.4-14.2)	11.7 (11.5-12.0)	12.6 (12.4-12.8)
Comorbidities					
Hypertension	22.2 (21.4-23.0)	36.2 (35.6-36.9)	20.7 (20.3-21.2)	30.4 (30.1-30.7)	29.3 (29.0-29.5)
Diabetes	7.7 (7.3-8.2)	11.6 (11.2-11.9)	9.1 (8.8-9.4)	8.2 (8.1-8.4)	8.7 (8.6-8.8)
Prior stroke/MI	2.7 (2.4-3.3)	5.4 (5.2-5.7)	3.1 (2.9-3.3)	6.0 (5.9-6.1)	5.3 (5.2-5.4)
Cancer	3.1 (2.9-3.4)	4.2 (4.0-4.4)	2.9 (2.8-3.1)	10.6 (10.4-10.7)	8.3 (8.2-8.4)
Emphysema/chronic bronchitis	1.8 (1.6-2.0)	4.6 (4.4-4.8)	2.8 (2.6-2.9)	5.8 (5.6-5.9)	5.0 (4.9-5.1)
Current smoker	9.6 (9.1-10.1)	18.8 (18.3-19.3)	12.5 (12.2-12.9)	19.6 (19.3-19.9)	18.0 (17.8-18.2)
Obesity (BMI ≥30)	9.7 (9.2-10.3)	37.5 (36.9-38.1)	30.9 (30.3-31.4)	27.0 (26.8-27.3)	28.0 (27.7-28.2)

Abbreviations: BMI, body mass index (calculated as weight in kilograms divided by height in meters squared); GED, general equivalency diploma; MI, myocardial infarction.

^a^ Unless otherwise indicated, data are expressed as weighted percentage of participants (95% CI).

^b^ Annual household income was categorized relative to the respective year’s federal poverty level from the US Census Bureau into middle to high income (≥200%) and low income (<200%). The weighted proportion of individuals with annual income at less than 200% of the federal poverty level was estimated using multiple imputation.

^c^ Based on where the housing unit of the survey participant was located. The 4 regions correspond to the regions recognized by the US Census Bureau.

### Temporal Trends From 2004 to 2018

#### Short Sleep Duration

**Figure 1.**
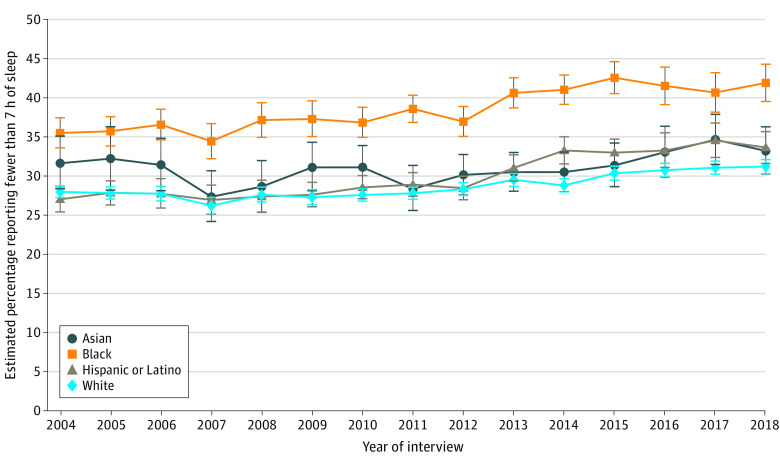
Overall Annual Estimated Prevalence of Short Sleep Duration by Race and Ethnicity Among US Adults Short sleep duration was defined as self-reported sleep duration of fewer than 7 hours in a 24-hour period (data source: National Health Interview Survey, 2004-2018). Annual prevalence estimates were obtained using multinomial logistic regression adjusted by age and US region (details are found in the Methods section and the eMethods in the Supplement). Error bars represent 95% CIs.

In 2004, the age- and region-adjusted estimated prevalence of short sleep (<7 hours) was 31.4% (95% CI, 28.1%-34.8%) among Asian individuals, 35.3% (95% CI, 33.4%-37.2%) among Black individuals, 27.0% (95% CI, 25.4%-28.6%) among Hispanic or Latino individuals, and 27.8% (95% CI, 27.1%-28.6%) among White individuals (Figure 1). From 2004 to 2018, the prevalence of short sleep increased significantly among Black (6.39 [95% CI, 3.32-9.46] percentage points), Hispanic or Latino (6.61 [95% CI, 4.03-9.20] percentage points), and White (3.22 [95% CI, 2.06-4.38] percentage points) individuals regardless of sex or household income stratum (*P* < .001 for each; Table 2 and eTable 1 in the Supplement). In the same period, the difference between Hispanic or Latino and White individuals increased significantly (3.39 [95% CI, 0.56-6.23] percentage points; *P* = .02) but did not change significantly between the other subgroups. In 2018, compared with the estimated prevalence among White individuals (31.0% [95% CI, 30.1%-31.9%]), short sleep duration among Black and Hispanic or Latino individuals was higher by 10.68 percentage points (95% CI, 8.12-13.24 percentage points; *P* < .001) and 2.44 percentage points (95% CI, 0.23-4.65 percentage points; *P* = .03), respectively (Table 2). The observed disparities between Black and White individuals remained in our sensitivity analysis that accounted for differences in overestimation of sleep duration between the 2 groups (eFigure 3 in the Supplement).

**Table 2.** Change in Short and Long Sleep Duration Prevalence by Race and Ethnicity, 2004 to 2018^a^

Characteristic	Participant race and ethnicity							
	Asian		Black		Hispanic or Latino		White	
	Change, percentage points (95% CI)	*P* value	Change, percentage points (95% CI)	*P* value	Change, percentage points (95% CI)	*P* value	Change, percentage points (95% CI)	*P* value
**Short sleep duration**								
Absolute change in prevalence, 2004-2018								
All	1.58 (−2.95 to 6.10)	.50	6.39 (3.32 to 9.46)	<.001	6.61 (4.03 to 9.20)	<.001	3.22 (2.06 to 4.38)	<.001
Women	4.02 (−2.35 to 10.40)	.22	7.07 (3.07 to 11.07)	<.001	4.44 (1.10 to 7.77)	.009	3.69 (2.13 to 5.26)	<.001
Men	−0.84 (−7.40 to 5.72)	.80	5.67 (1.20 to 10.14)	.01	8.75 (4.84 to 12.66)	<.001	2.72 (1.12 to 4.31)	.001
Low household income	3.24 (−6.94 to 13.41)	.53	8.14 (3.57 to 12.72)	<.001	6.57 (2.77 to 10.38)	<.001	5.02 (2.65 to 7.39)	<.001
Middle to high household income	0.98 (−4.24 to 6.20)	.71	4.97 (0.77 to 9.18)	.02	6.04 (2.33 to 9.75)	.001	2.96 (1.63 to 4.28)	<.001
Difference compared with White individuals, 2004								
All	3.65 (0.20 to 7.09)	.04	7.51 (5.45 to 9.57)	<.001	−0.95 (−2.73 to 0.82)	.29	NA	NA
Women	2.73 (−2.16 to 7.62)	.27	8.69 (5.97 to 11.41)	<.001	0.40 (−1.84 to 2.63)	.73	NA	NA
Men	4.40 (−0.59 to 9.39)	.08	6.18 (3.21 to 9.16)	<.001	−2.36 (−4.87 to 0.15)	.07	NA	NA
Low household income	1.49 (−6.30 to 9.28)	.71	1.49 (−1.59 to 4.56)	.34	−6.06 (−8.69 to −3.42)	<.001	NA	NA
Middle to high household income	4.24 (0.33 to 8.16)	.03	10.23 (7.31 to 13.16)	<.001	1.54 (−1.16 to 4.25)	.26	NA	NA
Difference compared with White individuals, 2018								
All	2.00 (−1.15 to 5.16)	.21	10.68 (8.12 to 13.24)	<.001	2.44 (0.23 to 4.65)	.03	NA	NA
Women	3.06 (−1.31 to 7.43)	.17	12.07 (8.74 to 15.39)	<.001	1.14 (−1.79 to 4.06)	.45	NA	NA
Men	0.84 (−3.71 to 5.39)	.72	9.14 (5.44 to 12.84)	<.001	3.68 (0.28 to 7.07)	.03	NA	NA
Low household income	−0.30 (−7.26 to 6.66)	.93	4.61 (0.47 to 8.74)	.03	−4.51 (−8.14 to −0.88)	.02	NA	NA
Middle to high household income	2.27 (−1.52 to 5.89)	.23	12.25 (8.95 to 15.55)	<.001	4.62 (1.76 to 7.49)	.002	NA	NA
Absolute change in difference compared with White individuals, 2004-2018								
All	−1.64 (−6.32 to 3.03)	.49	3.17 (−0.11 to 6.46)	.06	3.39 (0.56 to 6.23)	.02	NA	NA
Women	0.33 (−6.23 to 6.89)	.92	3.38 (−0.91 to 7.68)	.12	0.74 (−2.94 to 4.43)	.69	NA	NA
Men	−3.56 (−10.31 to 3.20)	.30	2.95 (−1.79 to 7.70)	.22	6.04 (1.81 to 10.26)	.005	NA	NA
Low household income	−1.79 (−12.24 to 8.66)	.74	3.12 (−2.03 to 8.27)	.24	1.55 (−2.94 to 6.03)	.50	NA	NA
Middle to high household income	−1.97 (−7.36 to 3.41)	.47	2.02 (−2.39 to 6.43)	.37	3.08 (−0.86 to 7.02)	.13	NA	NA
**Long sleep duration**								
Absolute change in prevalence, 2004-2018								
All	−0.12 (−1.84 to 1.60)	.89	−1.24 (−2.68 to 0.20)	.09	−1.42 (−2.52 to −0.32)	.01	0.17 (−0.34 to 0.68)	.51
Women	−0.81 (−3.42 to 1.80)	.54	−0.51 (−2.32 to 1.30)	.58	−2.05 (−3.46 to −0.64)	.004	0.15 (−0.43 to 0.74)	.61
Men	0.44 (−1.61 to 2.48)	.68	−2.13 (−4.24 to −0.02)	.05	−0.78 (−2.41 to 0.89)	.36	0.28 (−0.42 to 0.97)	.44
Low household income	−2.30 (−6.81 to 2.21)	.32	−2.12 (−4.65 to 0.41)	.10	−1.39 (−3.01 to 0.24)	.09	0.72 (−0.47 to 1.91)	.24
Middle to high household income	0.78 (−0.80 to 2.36)	.34	−0.51 (−2.17 to 1.16)	.55	−1.28 (−2.88 to 0.32)	.12	0.18 (−0.34 to 0.69)	.50
Difference compared with White individuals, 2004								
All	−0.92 (−2.41 to 0.56)	.22	2.90 (1.81 to 3.99)	<.001	1.09 (0.33 to 1.85)	.005	NA	NA
Women	−0.11 (−2.35 to 2.13)	.92	2.49 (1.17 to 3.80)	<.001	1.54 (0.46 to 2.61)	.005	NA	NA
Men	−1.72 (−3.48 to 0.04)	.06	3.41 (1.69 to 5.12)	<.001	0.66 (−0.46 to 1.79)	.25	NA	NA
Low household income	−0.06 (−4.20 to 4.08)	.98	3.49 (1.52 to 5.45)	<.001	−0.34 (−1.57 to 0.90)	.59	NA	NA
Middle to high household income	−1.40 (−2.61 to −0.18)	.02	1.47 (0.26 to 2.68)	.02	1.03 (−0.14 to 2.21)	.09	NA	NA
Difference compared with White individuals, 2018								
All	−1.27 (−2.25 to −0.29)	.01	1.44 (0.39 to 2.48)	.007	−0.55 (−1.47 to 0.36)	.24	NA	NA
Women	−1.07 (−2.55 to 0.40)	.15	1.83 (0.45 to 3.20)	.009	−0.67 (−1.74 to 0.41)	.23	NA	NA
Men	−1.56 (−2.81 to −0.30)	.02	1.00 (−0.41 to 2.42)	.16	−0.39 (−1.81 to 1.03)	.59	NA	NA
Low household income	−3.08 (−5.23 to −0.94)	.005	0.65 (−1.34 to 2.64)	.52	−2.45 (−4.04 to −0.85)	.003	NA	NA
Middle to high household income	−0.79 (−1.93 to 0.35)	.17	0.79 (−0.46 to 2.05)	.22	−0.42 (−1.62 to 0.78)	.49	NA	NA
Absolute change in difference compared with White individuals, 2004-2018								
All	−0.34 (−2.12 to 1.43)	.70	−1.46 (−2.98 to 0.05)	.06	−1.65 (−2.84 to −0.45)	.007	NA	NA
Women	−0.96 (−3.64 to 1.71)	.48	−0.66 (−2.56 to 1.24)	.50	−2.20 (−3.72 to −0.68)	.005	NA	NA
Men	0.16 (−2.00 to 2.32)	.88	−2.40 (−4.63 to −0.18)	.03	−1.05 (−2.86 to 0.76)	.25	NA	NA
Low household income	−3.02 (−7.68 to 1.64)	.20	−2.84 (−5.64 to −0.04)	.05	−2.11 (−4.12 to −0.10)	.04	NA	NA
Middle to high household income	0.61 (−1.06 to 2.27)	.47	−0.68 (−2.42 to 1.07)	.47	−1.45 (−3.13 to 0.23)	.09	NA	NA

Abbreviation: NA, not applicable.

^a^ Data source is the National Health Interview Survey from 2004 to 2018. Short sleep duration was defined as fewer than 7 hours of sleep in a 24-hour period; long sleep duration, as more than 9 hours of sleep in a 24-hour period. For change in prevalence and change in difference, positive percentage points indicate the prevalence (or its difference compared with White individuals) increased; negative percentage points, it decreased. Prevalence estimates were adjusted by age and region.

Similarly, the prevalence difference between Black women and White women persisted during the study period and was 12.07 percentage points (95% CI, 8.74-15.39 percentage points; *P* < .001) in 2018; among men in 2018, the difference was 9.14 percentage points (95% CI, 5.44-12.84 percentage points; *P* < .001). The prevalence difference between Hispanic or Latino men and White men, which was absent in 2004, increased and reached 3.68 percentage points (95% CI, 0.28-7.07 percentage points; *P* = .03) in 2018, whereas there was no significant change in the difference between women (1.14 [95% CI, −1.79 to 4.06] percentage points; *P* = .45) (Table 2 and eFigure 4 in the Supplement).

When stratified by income, there were no significant changes in the differences between groups during the study period. In 2018, the difference between Black individuals and White individuals was 12.25 percentage points (95% CI, 8.95-15.55 percentage points; *P* < .001) among those with middle to high income and 4.61 percentage points (95% CI, 0.47-8.74 percentage points; *P* = .03) among those with low income. In the same year, the difference between Hispanic or Latino individuals and White individuals was 4.62 percentage points (95% CI, 1.76-7.49 percentage points; *P* = .002) among those with middle to high income and −4.51 percentage points (95% CI, −8.14 to −0.88 percentage points; *P* = .02) among those with low income (Table 2). The differences in 2018 between Asian and White individuals were not significant for low (−0.30 [95% CI, −7.26 to 6.66] percentage points; *P* = .93) and middle to high (2.27 [95% CI, −1.52 to 5.89] percentage points; *P* = .23) income levels.

#### Long Sleep Duration

**Figure 2.**
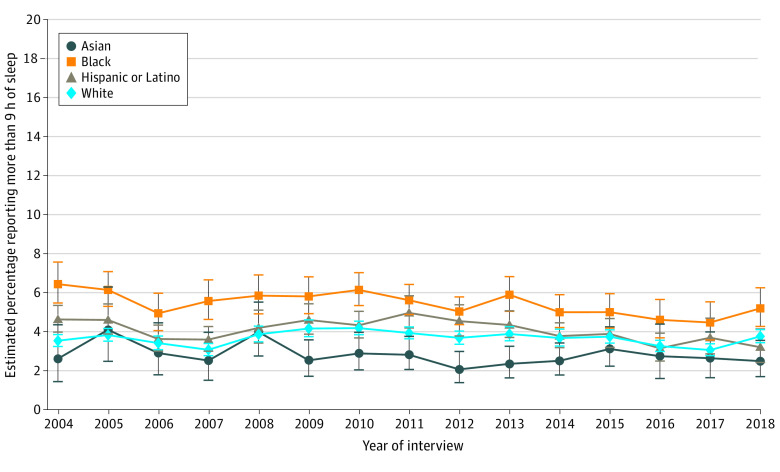
Overall Annual Estimated Prevalence of Long Sleep Duration by Race and Ethnicity Among US Adults Long sleep duration was defined as self-reported sleep duration of more than 9 hours in a 24-hour period (data source: National Health Interview Survey, 2004-2018). Annual prevalence estimates were obtained using multinomial logistic regression adjusted by age and US region (details are found in the Methods section and eMethods in the Supplement). Error bars represent 95% CIs.

In 2004, the adjusted estimated prevalence of long sleep (>9 hours) was 2.5% (95% CI, 1.4%-4.3%) among Asian individuals, 6.4% (95% CI, 5.4%-7.5%) among Black individuals, 4.6% (95% CI, 3.9%-5.3%) among Hispanic or Latino individuals, and 3.5% (95% CI, 3.2%-3.8%) among White individuals (Figure 2). From 2004 to 2018, the prevalence of long sleep significantly changed only among Hispanic or Latino individuals (−1.42 [95% CI, −2.52 to −0.32] percentage points; *P* = .01) (Table 2). In 2018, compared with the estimated long sleep prevalence among White individuals (3.7% [95% CI, 3.4%-4.1%]), prevalence was higher by 1.44 percentage points among Black individuals (95% CI, 0.39-2.48 percentage points; *P* = .007). Compared with White women, Black women had higher prevalence of long sleep during the study period (1.83 [95% CI, 0.45-3.20] percentage points; *P* = .009) (Table 2 and eFigure 5 in the Supplement). When stratified by income, the 2018 difference between Black and White individuals was not significant (low income, 0.65 [95% CI, −1.34 to 2.64] percentage points [*P* = .52]; middle to high income, 0.79 [95% CI, −0.46 to 2.05] percentage points [*P* = .22]) (eFigure 5 in the Supplement). When stratified by health status, the observed racial and ethnic disparities in short and long sleep duration persisted within each health status stratum during the study period (eTables 2 and 3 and eFigures 6 and 7 in the Supplement).

### Differences in the Association Between Sleep Duration and Age

#### Short Sleep Duration

**Figure 3.**
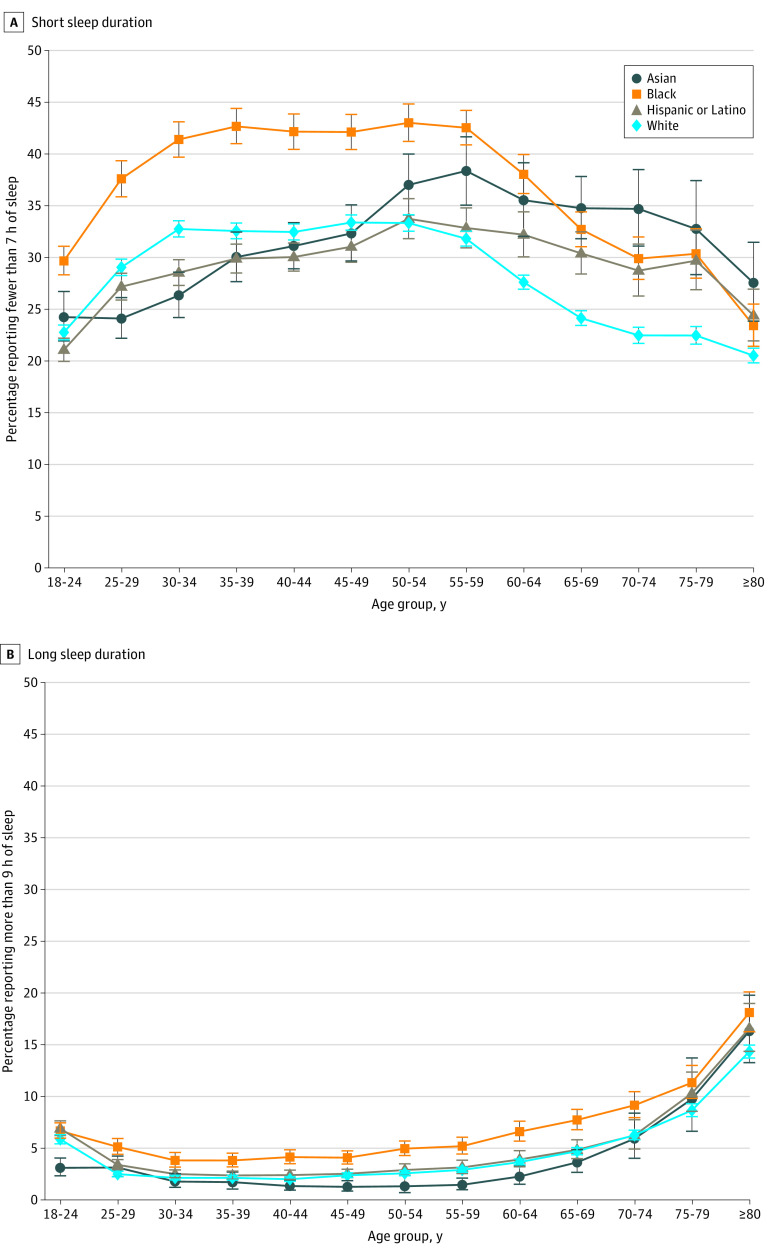
Association Between Age and Short and Long Sleep Duration by Race and Ethnicity Among US Adults Short sleep duration was defined as self-reported sleep duration of fewer than 7 hours in a 24-hour period; long sleep duration, self-reported sleep duration of more than 9 hours in a 24-hour period (data source: National Health Interview Survey, 2004-2018). Prevalence estimates for each age group were obtained using multinomial logistic regression (details are found in the Methods section and the eMethods in the Supplement). Error bars represent 95% CIs.

When compared with White individuals of the same age, short sleep duration was more prevalent among Black individuals, with a difference starting at 6.91 percentage points (95% CI, 5.35-8.46 percentage points) among those aged 18 to 24 years, peaking at 10.74 percentage points (95% CI, 8.92-12.55 percentage points) among those aged 50 to 59 years, and reaching 2.91 percentage points (95% CI, 0.76-5.10 percentage points) among those 80 years or older (Figure 3 and eFigure 8 in the Supplement). Among those older than 65 years, the prevalence of short sleep duration decreased for all subgroups as age increased. Similar patterns were observed by sex and among those with middle to high income (eFigure 9 in the Supplement).

#### Long Sleep Duration

Across all racial and ethnic groups, prevalence of long sleep was lower among those aged 30 to 60 years. Except for individuals aged 18 to 24 years, Black individuals had a higher prevalence than White individuals across all age groups, ranging from 1.69 percentage points (95% CI, 0.95-2.43 percentage points) among those aged 30 to 35 years to 3.78 percentage points (95% CI, 1.77-5.79 percentage points) among those 80 years or older (Figure 3). Similar patterns were observed by sex and income strata (eFigure 10 in the Supplement).

## Discussion

In this nationally representative sample of US adults from 2004 to 2018, we found an increasing prevalence of short sleep duration with persistence of racial and ethnic differences. Black individuals consistently had the highest prevalence of short sleep duration, reaching a difference of 10.68 percentage points compared with White individuals in 2018. The disparities were greater for Black women and Black individuals with middle to high income. In addition, the proportion of Hispanic or Latino individuals who reported short sleep increased among men, widening their gap with White men to 3.68 percentage points in 2018. Furthermore, Black individuals also had the highest prevalence of long sleep duration during the study period, although this disparity was narrower than that of short sleep. Prevalence among Asian individuals did not change significantly during the 15-year period and was not significantly different from that of White individuals. Notably, when analyzed by age, the racial and ethnic disparities were greatest among young and middle-aged Black adults and slightly narrowed among older Black adults.

This study expands the literature in several ways. First, we used data from 2004 to 2018 to describe trends in racial and ethnic disparities in sleep duration. Our findings regarding increasing prevalence of short sleep duration are consistent with those of previous NHIS studies,^11,12,31,32,33^ expanding them by quantifying the magnitude and significance of change in these racial and ethnic differences in the past 15 years and by analyzing disparities in long sleep duration. Of note, another study^34^ used data from the American Time Use Survey and found a slight increase in sleep duration from 2003 to 2016. Such a discrepancy may arise, in part, from how sleep duration is ascertained in each survey. In contrast with the holistic assessment of mean sleep length in the NHIS, the American Time Use Survey is a telephone-based survey that asks individuals to describe how they spent their day, starting at 4 am the previous day and ending at 4 am on the interview day.^35^ In addition, in the American Time Use Survey, self-reported time lying in bed may be recorded as sleep even if awake.^36^ Further research is needed to better understand this discrepancy. Second, we assessed the racial and ethnic differences in the association between each of these sleep duration outcomes and age. To the best of our knowledge, this has not been described previously. Third, we stratified our findings by sex and income, providing further insight into the characterization of these disparities. Fourth, we stratified by health status and found persistence in the disparities. Finally, we included Asian individuals in our analyses, finding that their estimates remained stable, without substantial differences compared with White individuals in 2018.

To understand why short sleep duration may be more common among Black individuals, it is important to discuss the influence of psychosocial stressors, such as race-based discrimination, on sleep health. The stress from perceived race-based discrimination (and its anticipation or vigilance) has been reported to contribute to shorter sleep duration,^37^ and Black individuals in the US are more likely to experience this than individuals of other racial or ethnic groups. We showed that the disparity in short sleep duration remained stable for 15 years for Black individuals. Further, it has been reported that the effect of perceived discrimination on sleep duration is greater among Black women than among Black men,^38^ which could partially explain our finding that the racial gaps in short sleep were the widest among Black women. Additional studies are needed to understand how these stressors derived from racial discrimination have changed over the last decades. Future work should also explore the extent to which Hispanic or Latino men may be facing increasing race- and ethnicity-based discrimination or other social stressors that could explain their widening gap with White men during the study period.

Long sleep was also persistently more prevalent among Black individuals, particularly among Black women. This finding may be explained by persistent racial differences in prevalence and type of underlying health conditions and socioeconomic stressors that could potentially lead to long sleep duration, including multimorbidity profiles (an indicator of multiple concurrent chronic conditions) and unemployment.^39,40,41^ Disparities in multimorbidity prevalence and unemployment rates persisted during the study period for Black individuals,^42,43^ which could support this explanation. However, further research is needed to understand these patterns and their causes.

The fact that the disparities were the widest among young and middle-aged adults suggests that factors related to working or employment conditions might disproportionally prevent Black individuals from having adequate sleep.^44^ Notably, when analyzed over years and by age, the gap between Black and White individuals with low income was substantially narrower compared with the gap among those with middle or high income. This finding suggests that a higher income may prevent White individuals from experiencing sleep duration alterations but does not have such a protective association among Black individuals. This differential association of income with sleep health is consistent with observations that higher educational attainment and professional responsibility are associated with lower odds of short sleep among White adults and with greater odds among Black and Hispanic or Latino adults.^45,46,47^ The findings of the present study suggest that income may be an indicator of educational and professional attainment and that Black individuals with higher income may be more commonly exposed to stressors preventing adequate sleep, including higher levels of racial discrimination.^48^

Our findings have important public health implications. These persistent disparities may contribute to other persistent racial and ethnic disparities in health. One study^26^ indicated that from 1999 to 2018, Black individuals had the highest prevalence of poor or fair health. Although the cross-sectional nature of the previous study^26^ and our study prevents us from assessing causality, the combined findings suggest that short or long sleep duration may be associated with detriments in health. Although the underlying cause of each sleep duration alteration may differ, both short and long sleep duration put individuals at increased risk of depression, reduced quality of life, cardiovascular disease, diabetes, and death, among other conditions.^7,49,50,51,52,53^ Such a persistent disparity in sleep duration among Black individuals may thus be associated with other health disparities and may serve as an imperfect indicator of overall disparities in health and well-being. For the national objective of achieving health equity, understood as the assurance of the condition of optimal health for all individuals,^54^ it is thus instrumental to also strive for the elimination of socioeconomic and health conditions that prevent racial and ethnic minority individuals from achieving adequate sleep.

Our findings also have important implications for the design of public health interventions, suggesting that targeted efforts should be made to improve sleep health among Black and Hispanic or Latino individuals. The observed persistent—and growing—disparities in sleep duration serve as an additional indicator of the consequences of the artificial hierarchy in which racial and ethnic minority individuals encounter higher barriers to maintaining a healthy life, including income distribution inequality, racial segregation, restricted access to medical care, and exposure to social and environmental conditions that affect health and sleep (eg, light, noise, and air pollution). Thus, and as with other disparities, public policies may be ineffective at eliminating these racial and ethnic disparities in sleep duration without accounting for systemic racism as a fundamental cause.

### Limitations

This study has several limitations. We relied on self-reported duration of sleep, which may be subject to recall and social desirability bias. Of note, across racial and ethnic groups, self-reported sleep has shown a low-to-moderate agreement with objective measurement of sleep duration.^29,55,56,57^ When compared with polysomnographic findings, White individuals overestimated their sleep duration by a mean of 73 minutes, whereas Black individuals overestimated it by 54 minutes.^29^ Such an overestimation may misclassify some participants’ sleep duration. Nonetheless, the potential 20-minute difference in self-reported sleep duration accuracy between White and Black individuals would only minimally explain the disparity between them, as suggested by our sensitivity analysis. Furthermore, self-reported sleep duration has important health implications, including consistent association with mortality across different populations^49,58,59,60,61^ and across racial and ethnic groups in the US.^62^ In addition, for the entire study period, we lacked other information that may have provided a more in-depth understanding of these disparities in sleep health, including subjective sleep quality, efficiency, and timing.^63^ Last, it is possible that the declining NHIS response rates may have influenced our findings. Nonetheless, the NHIS design has several strategies to mitigate nonresponse bias (eMethods in the Supplement).

## Conclusions

In this cross-sectional study of NHIS data from 2004 to 2018, there were significant differences in sleep duration by race and ethnicity, and the prevalence of unrecommended sleep duration was persistently higher among Black individuals. The disparities were greatest for Black women, Black individuals who had middle or high income, and young and middle-aged Black adults. Given the importance of sleep to health, the prevalence of short and long sleep duration may be associated with health disparities.

## Notes

Supplement. **eMethods.** Data Collection and Statistical Analysis
eReferences
**eTable 1.** Annualized Rate of Change in Short and Long Sleep Duration by Race and Ethnicity, 2004 to 2018 **eTable 2.** Change in Short Sleep Duration Prevalence by Race and Ethnicity and Health Status, 2004 to 2018 **eTable 3.** Change in Long Sleep Duration Prevalence by Race and Ethnicity and Health Status, 2004 to 2018 **eFigure 1.** Study Population Flowchart **eFigure 2.** Unadjusted Sleep Duration Distribution by Race and Ethnicity **eFigure 3.** Short Sleep Sensitivity Analysis Accounting for Differences in Bias of Self-reported Sleep Duration Between Black Individuals and White Individuals **eFigure 4.** Annual Estimated Prevalence of Short Sleep Duration by Race and Ethnicity Among US Adults Stratified by Sex and Income Level **eFigure 5.** Annual Estimated Prevalence of Long Sleep Duration by Race and Ethnicity Among US Adults Stratified by Sex and Income Level **eFigure 6.** Adjusted Annual Prevalence of Short Sleep Duration by Race and Ethnicity Among Those With Poor or Fair Health and With Good to Excellent Health, 2004 to 2018 **eFigure 7.** Adjusted Annual Prevalence of Long Sleep Duration by Race and Ethnicity Among Those With Poor or Fair Health and With Good to Excellent Health, 2004 to 2018 **eFigure 8.** Racial and Ethnic Differences in the Association Between Age and Sleep Duration **eFigure 9.** Association Between Age and Short Sleep Prevalence by Race and Ethnicity Stratified by Sex and Income Level **eFigure 10.** Association Between Age and Long Sleep Prevalence by Race and Ethnicity Stratified by Sex and Income Level
